# The epidemiology of rare types of hepatobiliary and pancreatic cancer from national cancer registry

**DOI:** 10.1007/s00535-022-01920-5

**Published:** 2022-09-26

**Authors:** Tomoyuki Satake, Chigusa Morizane, Ryoko Rikitake, Takahiro Higashi, Takuji Okusaka, Akira Kawai

**Affiliations:** 1 Department of Hepatobiliary and Pancreatic Oncology, National Cancer Center Hospital, 5-1-1, Tsukiji, Chuo-ku, Tokyo, 104-0045 Japan; 2 Rare Cancer Center, National Cancer Center, Tokyo, Japan; 3 Institute for Cancer Control, Division of Health Services Research, National Cancer Center, Tokyo, Japan; 4 Division of Health Services Research, National Cancer Center, Tokyo, Japan; 5 Department of Musculoskeletal Oncology and Rehabilitation, National Cancer Center Hospital, Tokyo, Japan; 6 Department of Hepatobiliary and Pancreatic Oncology, National Cancer Center Hospital East, Chiba, Japan

**Keywords:** Epidemiology, Hepatobiliary cancer, Pancreatic cancer, Rare tumors

## Abstract

**Background:**

Information on rare hepatobiliary and pancreatic (HBP) subtypes of cancer is scarce. We aimed to elucidate the incidence and clinical features of rare tumors in Japan using the National Cancer Registry (NCR), a new nationwide integrated population-based registry.

**Methods:**

The data of patients diagnosed in 2016–2017 were extracted from the NCR database, and classified by topography: liver cells, intrahepatic bile duct, gallbladder, extrahepatic bile duct, ampulla of Vater, and pancreas. Data were described and analyzed using the World Health Organization and General Rules tumor classifications. The incidences for all rare tumors including hepatoblastoma and adenosquamous cell carcinoma were calculated as the number of new cases divided by the corresponding total person years.

**Results:**

The NCR data yielded 8,239 patients with rare HBP tumors between 2016 and 2017. The ratios of rare tumors to all cancer types were 0.5%, 0.7%, 3.9%, 1.6%, 0.8%, and 7.2% in the liver, intrahepatic bile duct, gallbladder, extrahepatic bile duct, ampulla of Vater, and pancreas, respectively. Rare tumors occurred more frequently in men, except for gallbladder tumors. The main tumor stage was localized in liver cells (42.4%) and the intrahepatic bile duct (51.6%); more patients were diagnosed in advanced stage with gallbladder (84.1%) and extrahepatic bile duct (74.4%) tumors. Approximately equal percentage of patients were diagnosed at designated cancer care hospitals (DCCHs) and non-DCCHs, whereas 60% to 70% patients received treatment at DCCHs.

**Conclusion:**

This is the first report to provide comprehensive information on the epidemiological status of rare HBP tumors in Japan by utilizing population-based NCR data.

**Supplementary Information:**

The online version contains supplementary material available at 10.1007/s00535-022-01920-5.

## Introduction

In hepatobiliary and pancreatic oncology, despite recent therapeutic advances, liver cancer (LC), biliary tract cancer (BTC), and pancreatic cancer (PC) remain highly lethal. LC is the sixth-most common cancer (905,677 incident cases) worldwide, and the third-leading cause of cancer-related death (830,180 deaths), whereas PC is the twelfth-most common cancer (495,773 incident cases) and the seventh-leading cause of cancer-related death (466,003 deaths) [1]. Regarding BTCs, including cancers of the gallbladder, extrahepatic bile duct, and ampulla of Vater, they are relatively rare and only gallbladder cancer data is shown in IARC Cancer Base. Gallbladder cancer is the 23rd-most common cancer (115,949 incident cases) and the 20th-leading cause of cancer-related death (84,695 deaths) [1]. However, we should consider that LC, BTC, and PC respectively consist of several histological subtypes including major subtypes such as hepatocellular carcinoma in LC and adenocarcinoma in BTC and PC. While major histological subtypes have been well-investigated in many large studies, little is known about uncommon histologic subtypes, such as hepatoblastoma and adenosquamous cell carcinoma, and no study has systematically analyzed the features of all these uncommon subtypes in the hepatobiliary and pancreatic region.

Monitoring the actual condition of cancer treatment is essential in improving the quality of cancer care. In Japan, the data from hospital-based cancer registries (HBCRs) of cancer care hospitals designated by the national government (designated cancer care hospitals [DCCHs]) has been collected by the National Cancer Center to have a clinical picture of cancer practice and treatment since 2007 [2, 3]. Epidemiological information, including the incidence and distribution of age, sex, and region were mainly estimated by HBCRs and organ-specific cancer registries, and comprehensive actual numbers were insufficient. Conversely, the National Cancer Registry (NCR) was launched in January 2016 according to the Cancer Registry Act to obtain accurate nationwide cancer data of diagnosed cases in all hospitals of the 47 prefectures in Japan. The NCR is a new nationwide integrated population-based cancer registry (PBCR) for accurate incidence statistics [4]. The Cancer Registry Act provides an excellent resource to elucidate the incidence and distribution of rare cancers and uncommon subtypes.

In this study, we aimed to comprehensively elucidate the incidence and clinical features of rare cancers and uncommon subtypes in hepatobiliary and pancreatic cancer using the NCR data in 2016 and 2017.

## Methods

### Data source

We extracted the data of uncommon subtypes in hepatobiliary and pancreatic cancer cases diagnosed in 2016 and 2017 from the NCR database in Japan. The Japan NCR is a coordinated system of PBCRs collecting incidence and survival data on cases reported from every hospital in Japan. The population data in Japan are obtained from the Ministry of Health, Labour and Welfare. The standard global population data were obtained from the World Health Organization (WHO) [5].

The NCR data in this study were independently created and processed in accordance with the relevant data sharing laws.

### Data collection

The Japan NCR classifies tumors according to the International Classification of Disease for Oncology, third edition. For the present study, we defined hepatobiliary and pancreatic cancer using International Classification of Disease for Oncology, third edition (ICD-O-3), with topography code C22.0 (Liver cell), C22.1 (Intrahepatic bile duct), C23.9 (Gallbladder), C24.0 (Extrahepatic bile duct), C24.1 (Ampulla of Vater), and C25 (Pancreas). All data were described and analyzed according to worldwide classification: the WHO Classification of Tumors: Digestive System 5th edition [6], and domestic classification: The General Rules for the Clinical and Pathological Study of Primary Liver Cancer 6th edition [7], General Rules for the Clinical and Pathological Studies on cancer of the biliary tract 6th edition [8], and General Rules for the Study of Pancreatic Cancer 7th edition [9]. We categorized the information by ICD-O-3 codes. The information was then compiled by applying WHO Classification and Japanese Classification of each organ. We focused on uncommon histological subtypes; therefore, the following common morphology types of each topography were excluded: 8170/3 (hepatocellular carcinoma not otherwise specified (NOS) in C22.0), 8160/3 (cholangiocarcinoma and adenocarcinoma in C22.1), 8140/3 (adenocarcinoma NOS in C23.9, C24.0, C24.1), 8260/3 (papillary adenocarcinoma in C23.9, C24.0, C24.1), 8211/3 (tubular adenocarcinoma in C23.9, C24.0, C24.1), and 8500/3 (duct adenocarcinoma NOS in C25). All the other morphology types were defined as uncommon subtypes. Eventually, we comprehensively included all patients with newly diagnosed primary hepatobiliary and pancreatic cancer of uncommon subtypes from January 1, 2016, to December 31, 2017. For each patient, we retrieved data regarding the following factors: sex, age, detection mode (screening, incidental, unknown/others), tumor stage (localized, regional lymph node metastasis, invasion to adjacent structures, distant metastasis, or unknown), primary treatment method (surgery, laparoscopic surgery, endoscopic treatment, radiation therapy, chemotherapy, or others), and hospital (primary diagnosis, surgery, or chemotherapy). In this study, DCCHs were defined as hospitals designated as cancer care hospitals by the Ministry of Health, Labour and Welfare in Japan as of April 2020.

### Data analysis

The total number of cancer cases in C22, C23, C24, and C25 were collected from the NCR data [10]. We calculated the incidence of all uncommon subtypes in the population as a crude number of new cases with rare tumors divided by the total number of individuals in the Japanese population in 2016. Age-adjusted incidence rates were analyzed using weighted proportions of corresponding age groups according to the 1985 Japan standard population or world standard population. Age-adjusted incidence rates of total gallbladder cancer (C23) and total cancer in other parts of the biliary tract (C24) were extracted from the NCR data but only from 2017 records because the records from 2016 were not published at the time of analysis of this study [10].

### Ethical considerations

This study conducted investigative research based on the Cancer Registry Act. According to the procedure stipulated by the law, the protocol was reviewed by the Data Utilization Committee of the National Cancer Registration Office. As per the research ethics guidelines in Japan, our study was exempted from an ethics review by our institutional review board.

## Results

The NCR data yielded 8,239 patients with uncommon subtypes of hepatobiliary and pancreatic cancers between 2016 and 2017, including 422 patients with liver, 605 with intrahepatic bile duct, 645 with gallbladder, 450 with extrahepatic bile duct, 243 with ampulla of Vater, and 5,874 with pancreatic tumors (Table 1). As an overall picture for each organ (sum of all histological subtypes), 82,163, 16,473, 29,019, and 81,598 patients had liver and intrahepatic bile duct cancer, gallbladder cancer, cancer in other parts of the biliary tract, and pancreatic cancer in the same period based on the NCR public data. The ratio of uncommon subtypes to all subtypes of each organ was 0.5%, 0.7%, 3.9%, 1.6%, and 7.2% in the liver, intrahepatic bile duct, gallbladder, extrahepatic bile duct, ampulla of Vater, and pancreas, respectively. The age-adjusted incidences of each primary tumor are shown in Table 1. The incidence of pancreatic primary tumors was higher (1.1 per 100,000 population in WHO model) than that of other hepatobiliary primary tumors.

**Table 1:** Incidence of rare tumors in hepatobiliary and pancreas between 2016 and 2017

	Newly diagnosed number in 2016 and 2017	Percentage to total	Adjusted incidence /100,000 population in 1985 Japanese model	Adjusted incidence /100,000 population in WHO (2000–2025) model
Liver and intrahepatic bile duct (C22) total	82,163	100.000%	14.677	11.214
Liver and intrahepatic bile duct (C22) uncommon subtypes	1027	1.250%	0.282	0.271
Liver cell (C22.0)	422	0.514%	0.158	0.171
Intrahepatic bile duct (C22.1)	605	0.736%	0.124	0.100
Gallbladder and other parts of biliary tract (C23-C24) total	45,492	100.000%	6.844	5.387
Gallbladder and other parts of biliary tract (C23-C24) uncommon subtypes	1338	2.941%	0.248	0.199
Gallbladder (C23) total	16,473	100.000%	*2.4	N/A
Gallbladder (C23.9) uncommon subtypes	645	3.915%	0.120	0.096
Other parts of biliary tract (C24) total	29,019	100.000%	*4.2	N/A
Extrahepatic bile duct (C24.0)	450	1.551%	0.076	0.061
Ampulla of Vater (C24.1)	243	0.837%	0.052	0.042
Pancreas (C25) total	81,598	100.000%	14.143	11.319
Pancreas (C25) uncommon subtypes	5874	7.199%	1.348	1.106
*NCR public data in 2017				

Table 2 presents a comparison of the characteristics between patients with uncommon subtypes for each primary tumor type. Uncommon subtypes in hepatobiliary and pancreatic cancers—excluding rare gallbladder cancers—presented more frequently in men, whereas rare tumors of the gallbladder presented slightly more frequently in women. The main detection mode of rare tumors was incidental detection in the intrahepatic bile duct (57.4%) and pancreas (49.3%), but symptomatic in the gallbladder (53.5%) and extrahepatic bile duct (66.4%). The disease stage at the time of diagnosis differed among primary sites. The main tumor stage was localized in LC, including liver cell (42.4%) and intrahepatic bile duct (51.6%), whereas more patients were diagnosed in the advanced stage, defined as invasion to adjacent structures and distant metastasis, in BTC, including gallbladder (84.1%), extrahepatic bile duct (74.4%), and ampulla of Vater (53.9%). Initial treatment was also different among the primary sites. For each primary site, over half of the patients were initially treated with surgery, including laparoscopic surgery, and this ratio was especially higher ratio for patients with rare tumors in the ampulla of Vater (73.3%). Contrastingly, higher ratios of patients with rare tumors in the gallbladder (43.3%) and liver cell (41.2%) were treated with chemotherapy. Radiation therapy was unusual as an initial treatment strategy for uncommon subtypes of hepatobiliary and pancreatic cancers.

**Table 2:** Characteristics of patients diagnosed with rare tumors in hepatobiliary and pancreas

	Liver cell (C22.0)		Intrahepatic bile duct (C22.1)		Gallbladder (C23.9)		Extrahepatic bile duct (C24.0)		Ampulla of Vater (C24.1)		Pancreas (C25)
	*n* = 422		*n* = 605		*n* = 645		*n* = 450		*n* = 243		*n* = 5874
Sex											
Male	254	60.2%	418	69.1%	315	48.8%	290	64.4%	145	59.7%	3310
Female	168	39.8%	187	30.9%	330	51.2%	160	35.6%	98	40.3%	2564
Detection											
Screening	30	7.1%	43	7.1%	22	3.4%	22	4.9%	28	11.5%	680
Incidental	173	41.0%	347	57.4%	266	41.2%	117	26.0%	96	39.5%	2897
Symptomatic	198	46.9%	195	32.2%	345	53.5%	299	66.4%	114	46.9%	2124
Autopsy/unknown	21	5.0%	20	3.3%	12	1.9%	12	2.7%	5	2.1%	173
Stage											
Localized	179	42.4%	312	51.6%	52	8.1%	56	12.4%	74	30.5%	2425
Regional lymph node metastasis	4	0.9%	43	7.1%	15	2.3%	13	2.9%	22	9.1%	201
Invasion to adjacent structures	39	9.2%	80	13.2%	310	48.1%	258	57.3%	96	39.5%	1512
Distant metastasis	95	22.5%	112	18.5%	232	36.0%	77	17.1%	35	14.4%	1343
Unknown	105	24.9%	58	9.6%	36	5.6%	46	10.2%	16	6.6%	393
Primary treatment											
Surgery	191	45.3%	302	49.9%	290	45.0%	247	54.9%	172	70.8%	3170
Laparoscopic surgery	26	6.2%	88	14.5%	77	11.9%	6	1.3%	6	2.5%	598
Endoscopic treatment	0	0.0%	2	0.3%	4	0.6%	12	2.7%	8	3.3%	18
Radiation therapy	13	3.1%	15	2.5%	10	1.6%	7	1.6%	1	0.4%	74
Chemotherapy	174	41.2%	151	25.0%	279	43.3%	155	34.4%	52	21.4%	2021
Others	51	12.1%	59	9.8%	18	2.8%	20	4.4%	4	1.6%	133

**Fig. 1:**
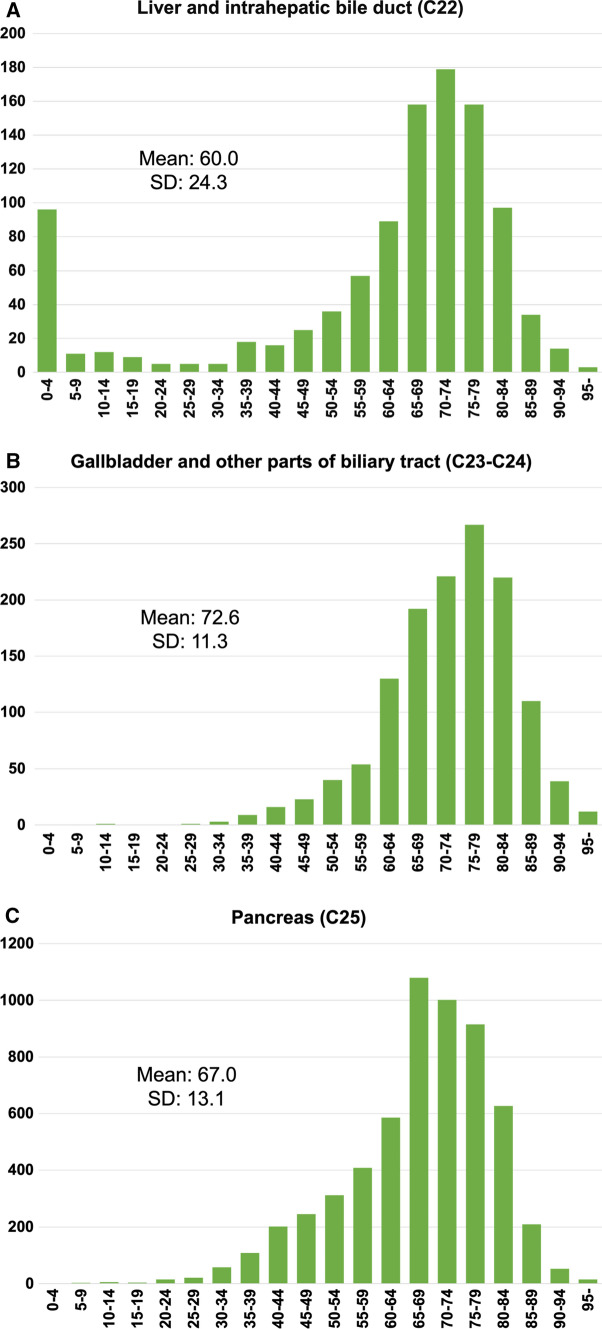
Age distribution (at diagnosis) of patients with rare tumors in the Japanese national cancer registry: **a** liver and intrahepatic bile duct; **b** gallbladder and other parts of biliary tract; **c** pancreas *SD* standard deviation

With regard to the age of onset, the most frequent age group varied between primary sites as follows: 70–74 years in liver and intrahepatic bile duct, 75–79 years in gallbladder and other parts of biliary tract, and 65–69 years in pancreas (Fig. 1). The histogram of age distribution of liver indicates a small peak or long tail in childhood, adolescent and young adult (AYA) with a larger standard deviation than biliary tract and pancreas. In patients under 20, the most common primary sites were liver and intrahepatic bile duct, and almost all patients with rare liver and intrahepatic bile duct cancers were diagnosed at 4 years of age or younger.

**Fig. 2:**
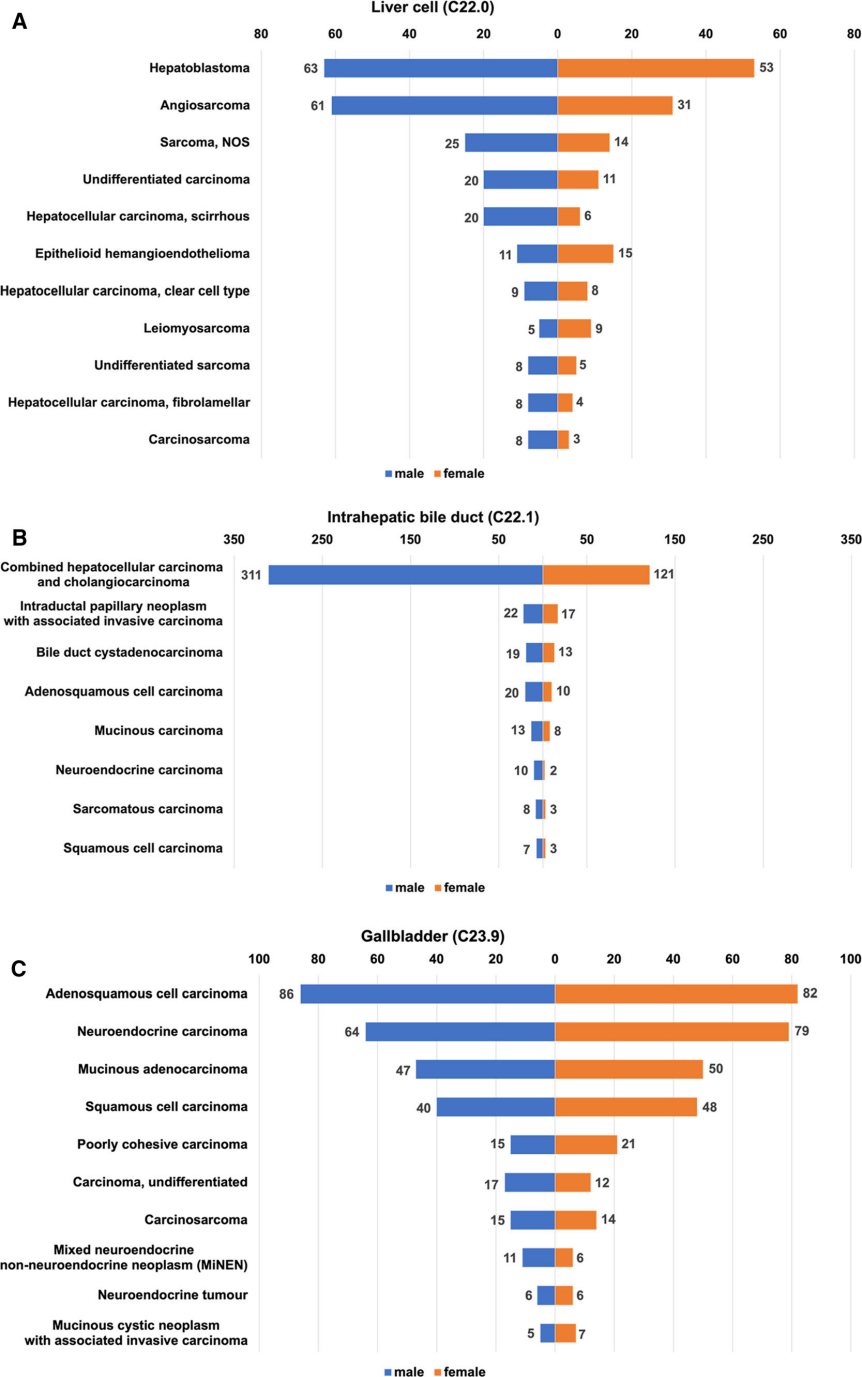

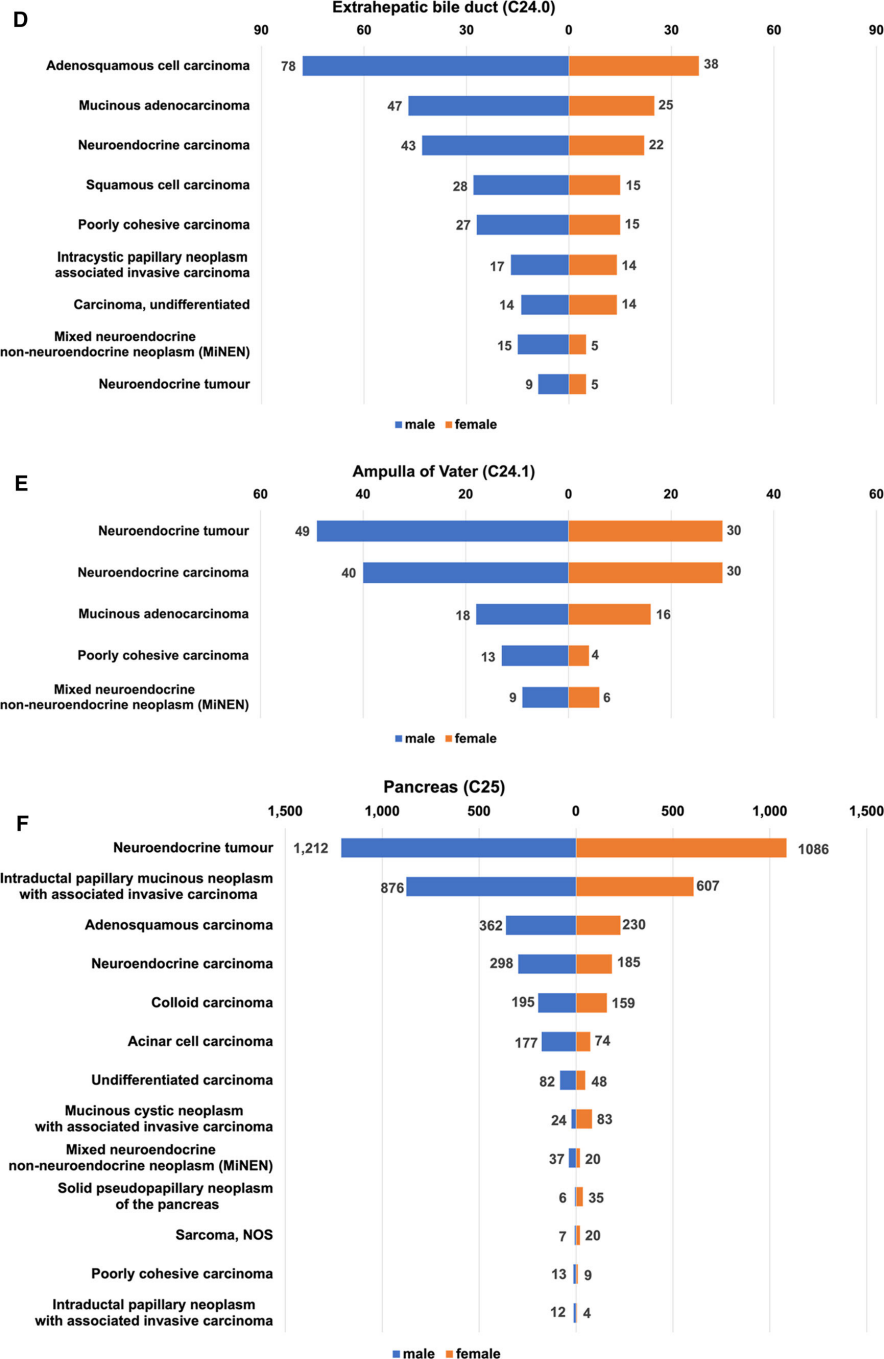
Crude number and sex distribution of patients with rare tumors between 2016 and 2017. Each rare tumor for 10 or more patients is listed by topography: **a** liver cell; **b** intrahepatic bile duct; **c** gallbladder; **d** extrahepatic bile duct; **e** ampulla of Vater; **f** pancreas

Information on sex-based differences and the number of each rare tumor was extracted only in cases where data on more than 10 patients was available, as shown in Fig. 2. Generally, the number of patients with uncommon subtypes of gallbladder cancer was similar between men and women, while the number of patients in uncommon subtypes of other organs was higher in men than in women. The number of patients with epithelioid hemangioendothelioma, mucinous cystic neoplasm with associated invasive carcinoma, and solid pseudopapillary neoplasm was significantly higher in women than in men.

**Fig. 3:**
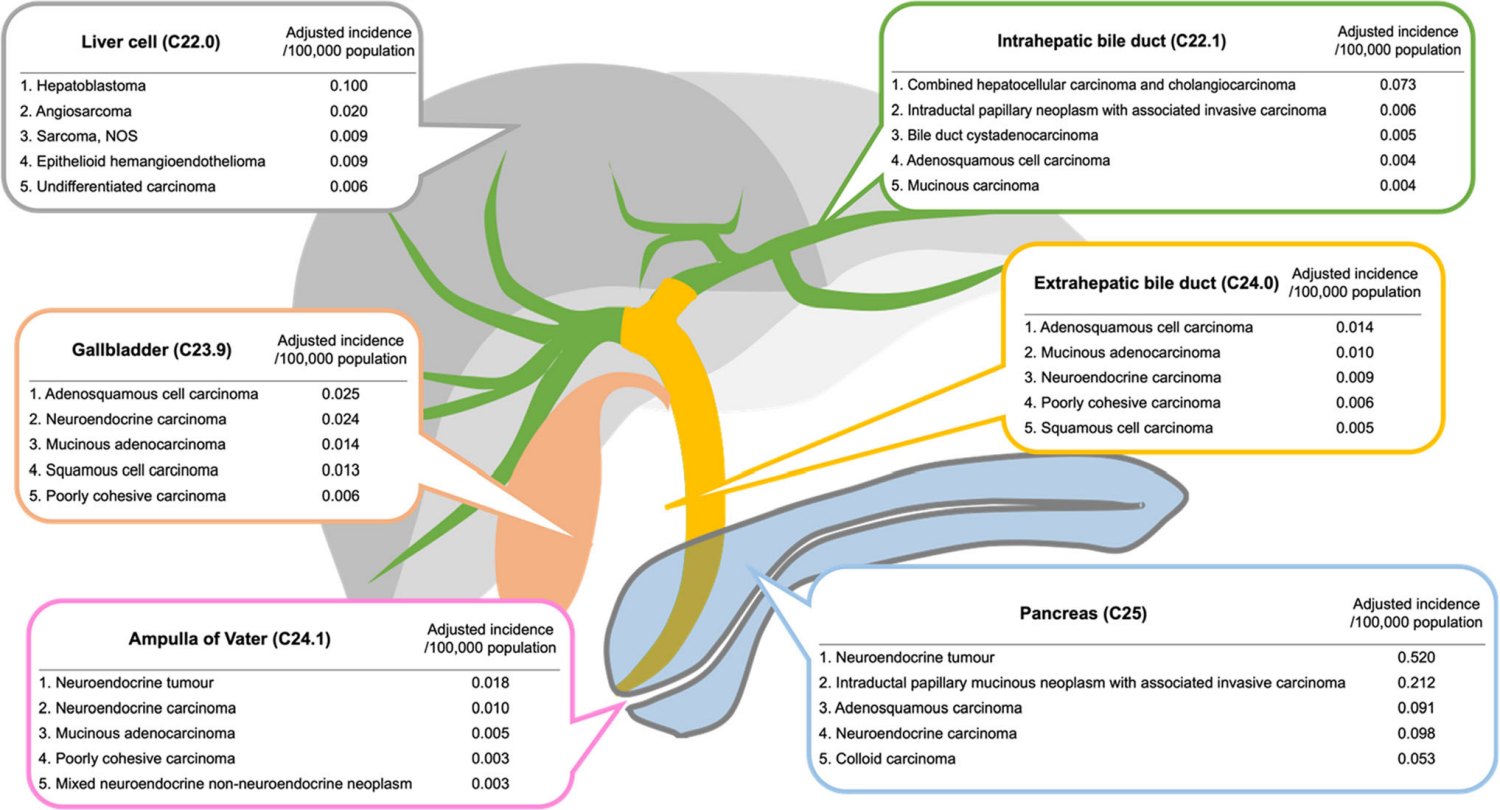
Representative rare tumors are illustrated in descending order of age-adjusted incidence in WHO (2000–2025) model

A summary of the top five most uncommon subtypes for each organ in descending order is shown in Fig. 3. Correspondence lists between rare tumors in this study and ICD-O-3 are shown in Supplementary Table S1 and are summarized in Supplementary Table S2-S7 as per the number of patients, incidence, and sex-related differences.

**Fig. 4:**
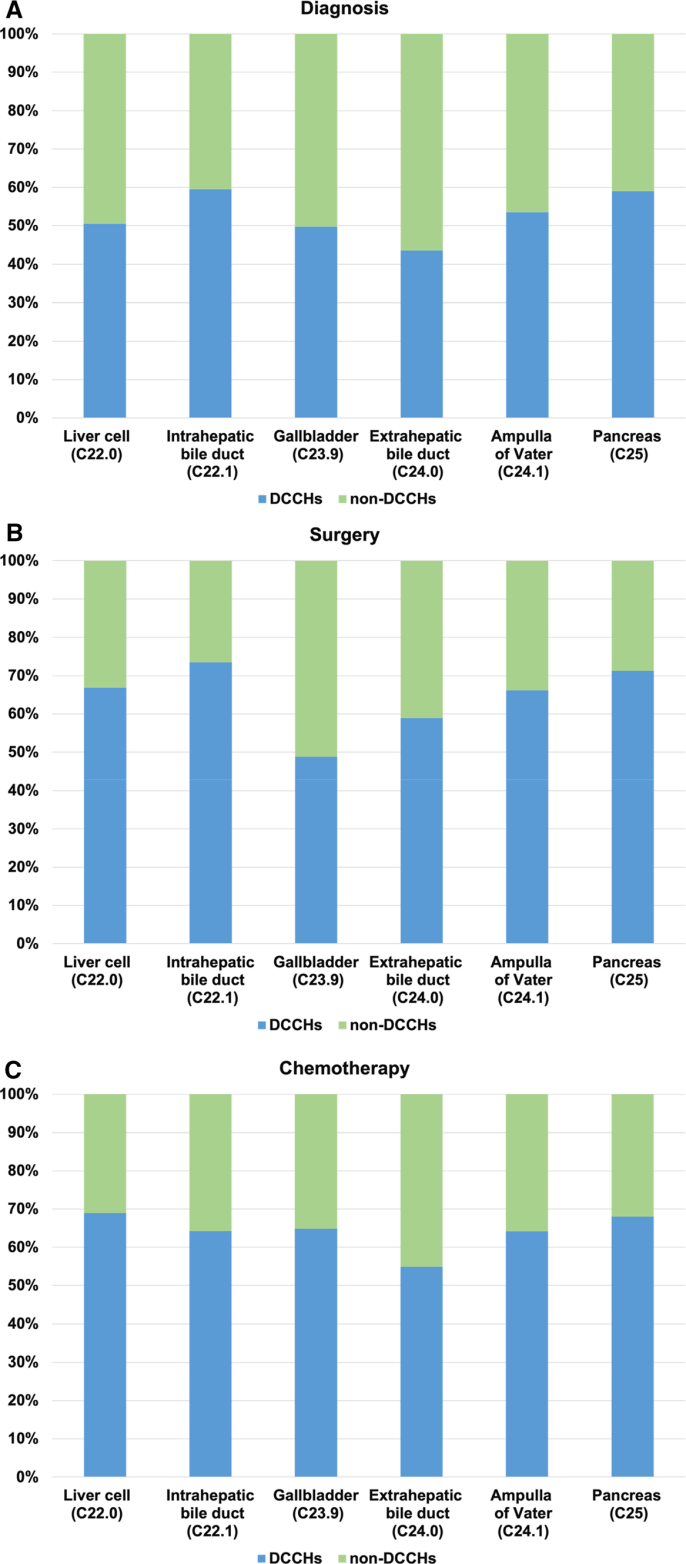
Difference in distribution of diagnosis and treatment (surgery and chemotherapy) of rare tumors between designated cancer care hospitals (DCCHs) and non-DCCHs in Japan: **a** diagnosis, **b** surgery, and **c** chemotherapy

The differences in diagnosis and treatment between DCCHs and non-DCCHs are shown in Fig. 4. The ratio of DCCHs and non-DCCHs were approximately half and half in diagnosis (43.6% in extrahepatic bile duct to 59.5% in intrahepatic bile duct), whereas about 60% to 70% more patients received treatment (surgery or chemotherapy) at DCCHs, except in cases of gallbladder cancer, wherein only 48.8% of patients underwent surgery at DCCHs.

## Discussion

To the best of our knowledge, this was the first study to comprehensively elucidate the incidence and clinical features of uncommon subtypes in hepatobiliary and pancreatic cancers. Except for the common histological subtypes, such as hepatocellular carcinoma, cholangiocarcinoma, and adenocarcinoma, all of uncommon subtypes were included in this study, using NCR data. There is no internationally agreed upon definition of rare cancers. In the USA, a recent analysis held at the National Cancer Institute in 2007 employed the definition of < 15 incident cases per 100,000 per year [11]. Conversely, the Surveillance of Rare Cancers in Europe (RARECARE) project proposed a list of rare cancers and developed a new incidence-based definition of < 6 incident cases per 100,000 per year [12]. Our study revealed that the incidence of uncommon subtypes was extremely rare and less than 0.3 per 100,000 population for hepatobiliary cancer and at most 1.1 per 100,000 population for pancreatic cancer. Moreover, these incidences easily met any suggested definition of rare cancers. Although there have been many studies about rare tumors, many of them were retrospective observational studies targeted at single rare tumor types on a limited scale [13, 14]. These studies could analyze practice treatment, but were inappropriate to obtain epidemiologic information. Recently, PBCRs data were established in many countries, and were used for the study of rare tumors [12, 15–17]. A European report used the RARECARE definition to hierarchically structure the list of rare tumors into three layers: families of tumors (tumors with the same referral pattern), clinically meaningful tumors (perceived by clinicians as single diseases), and WHO tumor entities. In this list, the first layer is marked with the number 1 (Tier 1), the second layer with 2 (Tier 2), and the WHO entities are marked with 3. A total of 190 types of tumors are classified as rare cancers, including nine types of Tier 1 tumors and 181 types of Tier 2 tumors [12]. Regarding rare hepatobiliary and pancreatic tumors, many of the uncommon subtypes covered in our study were included in the RARECARE lists. However, neuroendocrine tumors and sarcoma are not divided by each primary site, and the details of adenocarcinoma with variants are currently unclassified. We listed details of adenocarcinoma with variants, neuroendocrine neoplasms, and sarcoma. Furthermore, the primary sites of the intrahepatic bile duct and the ampulla of Vater were not included for “liver” or “biliary tract,” but divided individually. Thus, we believe that this study reveals a more comprehensive and detailed burden of rare tumors than previous reports.

When evaluating individual tumors, the incidence of several tumors differed from some previous reports. Fibrolamellar hepatocellular carcinoma is a rare variant of hepatocellular carcinoma, and usually found in the young (< 40 years) and in Caucasians without underlying hepatitis or cirrhosis. Fibrolamellar hepatocellular carcinoma accounts for 0.85% of all cases of primary liver cancer in the United States, and its incidence is 0.02 per 100,000 population in Europe [15, 18]. In Japan or in Asia, fibrolamellar hepatocellular carcinoma is rare [17]. This difference of incidence between Caucasians and Asians were similarly suggested in our study. Intraductal papillary mucinous neoplasm (IPMN) is a common lesion, with an estimated prevalence of 3–6% in the general population. When IPMN progresses to an invasive pancreatic ductal adenocarcinoma, it is referred to as “IPMN with an invasive carcinoma” or “intraductal papillary mucinous carcinoma” (IPMC). IPMC accounts for approximately 10% of resected pancreatic cancers of ductal origin; however, the incidence of IPMC remains ambiguous [19, 20]. From the previous reports, although the incidence of IPMC was 0.01 per 100,000 population in Europe, it was 0.14 per 100,000 population in Korea and 0.50 per 100,000 population in Japan [15, 17]. In our study, the crude incidence further increased to 0.58 per 100,000 population (Supplementary Table S6). Similarly, the incidence of undifferentiated carcinoma with osteoclast-like giant cells was higher than previous reports (< 0.01 per 100,000 population in Europe and Japan) [15, 17]. These differences indicate that the Japan NCR project could more comprehensively cover all rare tumors, whereas many differences may exist in the pathological diagnoses or real incidence between countries.

Furthermore, our study elucidated not only the burden, but also the clinical features of rare tumors, which were not fully covered in previous studies [12, 15–17]. Regarding the sex ratio, there were no major difference between common morphology types and rare tumors as a whole. As is well-known, liver cancer, extrahepatic bile duct cancer, and ampulla of Vater cancer present more frequently in men, whereas gallbladder cancers present more frequently in women [1, 21]. This trend was common in rare tumors. However, rare tumors of the intrahepatic bile duct or gallbladder were seen more frequently in men than common morphology type tumors. On the whole, based on our study, rare hepatobiliary and pancreatic tumors occurred more frequently in men. Individually considering each rare tumor, acinar cell carcinoma and combined hepatocellular carcinoma and cholangiocarcinoma were common in men, whereas mucinous cystic neoplasm with associated invasive carcinoma and solid pseudopapillary neoplasm were common in women, as previously reported [22, 23].

Many rare tumors of the extrahepatic bile duct were diagnosed via symptomatic reports, which indicated rare tumors may have similar characteristics to common morphology type because of tumor localization. However, only 17% of rare tumors of the extrahepatic bile duct were diagnosed with distant metastasis, which was less than reported in common morphology types [21]. Similarly, the tumors with distant metastasis were less frequent in intrahepatic bile duct and pancreas. These features indicate that some rare tumors have less aggressive characteristics than those of common morphology types.

In Japan, cancer treatment is provided through multiple facilities, rather than being centralized. According to an analysis using a hospital-based cancer registry in Japan, even rare cancers with an estimated annual incidence of one case per 100,000 individuals were treated at nearly 300 DCCHs [24]. Conversely, in some European countries, rare cancer treatment is centralized through a small number of large specialized institutions. For example, the number of hospitals providing 75% of treatments for rare liver cancers was 22 in Belgium (population, 10.5 million), 12 in Bulgaria (population, 7.7 million), and 36 in the Netherlands (population, 16.3 million) [15]. Our study revealed the real-world status of diagnosis and treatment of rare hepatobiliary and pancreatic tumors in Japan. There were no large differences in diagnosis between each primary site, which means roughly half of rare tumors were diagnosed in non-DCCHs (not specialized facilities). This is not only because of the distribution characteristics of cancer treatment facilities, but also because of difficulties of pre-treatment biopsies and diagnosis of rare tumors. Although we expected patients diagnosed with rare cancer to be referred to a DCCH for specialized treatment, only about 10% more patients received treatment at DCCHs in comparison with those who were diagnosed at DCCHs. This difference may be explained by the lack of system to centralize rare tumors treatment. Notably, patients with rare tumors of the gallbladder received surgery less frequently at DCCHs because of difficulties of pre-operative biopsy and diagnosis of rare tumors in gallbladder, or because the cancer was found incidentally after resection due to suspected cholecystitis. Since information on rare cancers is lacking, it is important to improve access for patients and facilitate communication between DCCHs and non-DCCHs. Low incidence is a major obstacle to conducting clinical trials to develop effective new treatments for rare tumors. One way to overcome this obstacle would be to centralize patients with rare tumors to DCCHs that excel in management and treatment of that specific cancer subtype.

This study had several limitations that require consideration when interpreting the results. First, because the registry started in 2016, we could only analyze data between 2016 and 2017. The number of uncommon cancers is small, leading to the unstability of the incidence data. Moreover, because the follow-up duration was still short, we do not yet have sufficient data on survival. We plan to solve these issues after we accumulate sufficiently mature data. Regarding the classification of rare tumors, we used both the worldwide WHO classification of tumors and domestic classification, as described in the latest versions as of 2016 [6–9]. However domestic classification (general rules) of LC, BTC, and PC have been revised, and there are some differences in pathological classification of rare tumors, such as neuroendocrine neoplasm. Finally, several important epidemiological factors that are recorded in HBCRs are not captured by the NCR. While HBCRs did not cover all the cancer cases in Japan, they prioritized the clinical information, including the classification of the Union for International Cancer Control (UICC) Tumor Node Metastasis of malignant tumor stage and treatment dates of each patient, which are not obtained by the NCR. Thus, an HBCR is superior to PBCR in facility comparison. However, the size of the present study compensates for this weakness to a great extent and provides a comprehensive epidemiological understanding of all rare hepatobiliary and pancreatic tumors.

In conclusion, we have provided comprehensive information on the epidemiological status on all rare hepatobiliary and pancreatic tumors in Japan by utilizing population-based NCR data for the first time. We plan to expand this study and analyze survival data and trends in mortality. Moreover, detailed information will be analyzed for each individual rare cancer in the future. The current study serves as an important and useful basis for understanding and developing treatment for rare tumors.

## Supplementary Information

Below is the link to the electronic supplementary material.

Supplementary file1 (XLSX 30 KB)
