# Correction: The epidemiology of rare types of hepatobiliary and pancreatic cancer from national cancer registry

**DOI:** 10.1007/s00535-022-01944-x

**Published:** 2022-12-23

**Authors:** Tomoyuki Satake, Chigusa Morizane, Ryoko Rikitake, Takahiro Higashi, Takuji Okusaka, Akira Kawai

**Affiliations:** 1 Department of Hepatobiliary and Pancreatic Oncology, National Cancer Center Hospital, 5-1-1, Tsukiji, Chuo-ku, Tokyo, 104-0045 Japan; 2 Rare Cancer Center, National Cancer Center, Tokyo, Japan; 3 Institute for Cancer Control, Division of Health Services Research, National Cancer Center, Tokyo, Japan; 4 Division of Health Services Research, National Cancer Center, Tokyo, Japan; 5 Department of Musculoskeletal Oncology and Rehabilitation, National Cancer Center Hospital, Tokyo, Japan; 6 Department of Hepatobiliary and Pancreatic Oncology, National Cancer Center Hospital East, Chiba, Japan

**Correction: Journal of Gastroenterology (2022) 57:890–901** 10.1007/s00535-022-01920-5

In the original publication of the article, the “Acknowledgements” section was omitted, and it is given in this correction as below:

**Acknowledgements** We thank the Rare Cancer Center at the National Cancer Center for giving us the opportunity to collaborate with appropriate researchers. This work was supported in part by The National Cancer Center Research and Development Fund (28-A-16 and 31-A-14).

The original article has been corrected.

